# Prognostic impact of multimorbidity in patients with type 2 diabetes and ST-elevation myocardial infarction

**DOI:** 10.18632/oncotarget.22324

**Published:** 2017-11-06

**Authors:** Bartosz Hudzik, Ilona Korzonek-Szlacheta, Janusz Szkodziński, Marek Gierlotka, Andrzej Lekston, Barbara Zubelewicz-Szkodzińska, Mariusz Gąsior

**Affiliations:** ^1^ Third Department of Cardiology, School of Medicine with the Division of Dentistry in Zabrze, Medical University of Silesia in Katowice, Silesian Center for Heart Disease, Zabrze, Poland; ^2^ Department of Nutrition-Related Disease Prevention, School of Public Health in Bytom, Medical University of Silesia in Katowice, Katowice, Poland

**Keywords:** myocardial infarction, diabetes mellitus, comorbidity, multimorbidity, prognosis

## Abstract

**Introduction:**

There is an increasing body of evidence on the clinical importance of multimorbidity, which is defined as the coexistence of two or more chronic conditions. Type 2 diabetes (T2DM) is one of the most frequent chronic conditions. Most adults with type 2 diabetes have at least 1 coexisting chronic condition and approximately 40% have 3 or more. Prior studies have suggested that cardiovascular (CVD) and non-CVD comorbid conditions yield worse outcomes in patients hospitalized with ST-elevation myocardial infarction (STEMI). It is unclear, however, the extent to which multimorbidity has a cumulative effect on long-term risk. Therefore we have set out to determine the prognostic value of multiple comorbidity on long-term outcomes in this population of patients.

**Methods:**

A total of 277 patients with T2DM and STEMI undergoing primary percutaneous coronary intervention (PCI) were enrolled. Based on the number of comorbidities the study population was divided into two groups: group 1 (N=58) with ≤ 1 comorbidity and group 2 (N=219) with ≥ 2 comorbidities.

**Results:**

Comorbid conditions were prevalent among study participants (Figure 1). The median number of comorbidities was three. 15.9% of patients had one comorbidity and 22.0%, 34.3%, and 22.7% of patients had two, three or at least four comorbid conditions respectively. A majority of patients had at least one CVD comorbidity (6.1% of patients had none), whereas 53.1% of patients did not have any non-CVD comorbidity. During hospitalization 3 out of 58 patients (5.2%) died in group 1 and 25 of 219 patients (11.4%) died in group 2. The number of comorbid conditions was not an independent predictor of in-hospital death. During 12-month follow-up, 5 of 58 patients (8.6%) and 42 of 219 patients (19.9%) died, respectively in group 1 and 2 (P=0.05). The number of comorbid conditions proved in ROC analysis that for 12-month mortality, the prognostic value was modest, but for 12-month acute coronary syndromes the prognostic value was good. Increase in the number of comorbid conditions by one was associated with a 15% increase in the relative risk of 12-month mortality and a 41% increase in the relative risk of 12-month acute coronary syndromes (ACS).

**Conclusions:**

Comorbid conditions are highly prevalent among these groups of patients. Majority of patients have at least 2 other cardiovascular comorbidities and one or two non-cardiovascular comorbidities. In terms of long-term follow-up, multimorbidity was associated with worse outcomes. The risk of both long-term mortality and ACS increased with the increasing number of comorbidities. In summary, our findings highlight the importance of indentifying patients with multimorbidity. This, in turn, could allow for provision of better care to these high-risk and complex group of patients.

## INTRODUCTION

There is an increasing body of evidence on the clinical importance of multimorbidity, which is defined as the coexistence of two or more chronic conditions [1]. The number of individuals with multiple concomitant chronic conditions has increased significantly during the past few decades [2, 3]. Type 2 diabetes mellitus (T2DM) is one of the most frequent chronic conditions. Most adults with T2DM have at least 1 coexisting chronic condition and approximately 40% have 3 or more [4, 5] On one hand, it is one of the most commonly measured diseases in studies of multimorbidity, on the other it is one of the most frequently detected conditions in multimorbid disease clusters [6, 7]. T2DM is a major contributor to the development of cardiovascular disease (CVD), stroke, chronic kidney disease, non-traumatic lower limb amputations, blindness, and depression. Growing number of chronic diabetes-related complications and comorbid conditions have been associated with poor metabolic control, less optimal disease management, higher health service utilization, impaired physical functioning, and worse outcomes [7–10].

There is an unequivocal predilection to coronary artery disease and ST-elevation myocardial infarction (STEMI) in older patients. This, in turn, carries a higher burden of additional comorbidities [11]. Prior studies have suggested that CVD and non-CVD comorbid conditions yield worse outcomes in patients hospitalized with acute myocardial infarction (AMI) [12–15]. Chronic conditions, e.g. diabetes mellitus, hypertension, atrial fibrillation among others each by themselves are associated with worse outcomes following STEMI. It is, however, unclear of the extent to which multimorbidity has a cumulative effect on long-term risk. Despite the important impact of multiple comorbidities, the practice of excluding patients with significant multimorbidity from clinical trials results in a lack of evidence with regard to management in this group. The odds of having multiple comorbidities increases significantly over time. As the number of patients with multiple comorbid conditions continues to rise, the need to develop strategies to manage these patients becomes increasingly important.

Risk stratification of patients with AMI at the time of initial presentation is important for their optimal management. Generally, prognostic scores do not contain any information on comorbidities as patients with multimorbidity are frequently excluded from randomized trials. To the best of our knowledge no studies have reported the prevalence of comorbidities and their impact on short- and long-term prognosis in patients with T2DM and STEMI. Therefore we have set out to determine the prognostic value of multiple comorbidity on long-term outcomes in this population of patients.

## RESULTS

**Figure 1:**
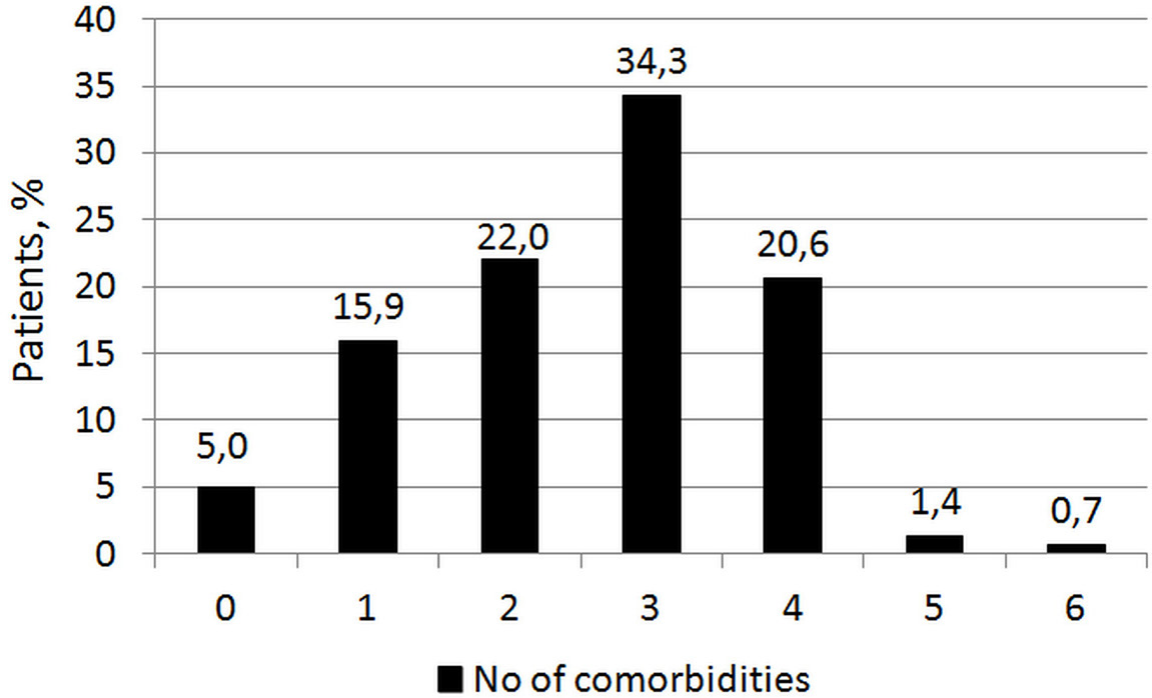
Distribution of the number of comorbid conditions in the whole study population

**Figure 2:**
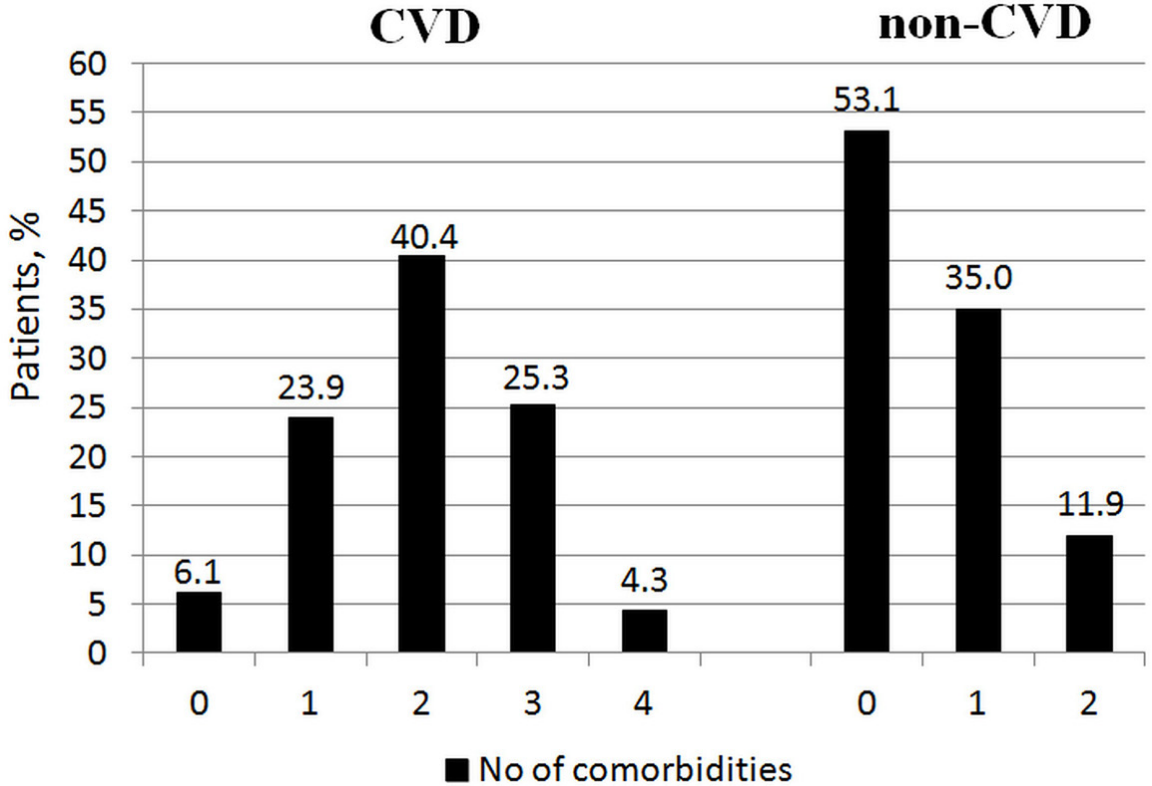
Distribution of the number of comorbid cardiovascular (CVD) and non-cardiovascular (non-CVD) conditions

**Figure 3:**
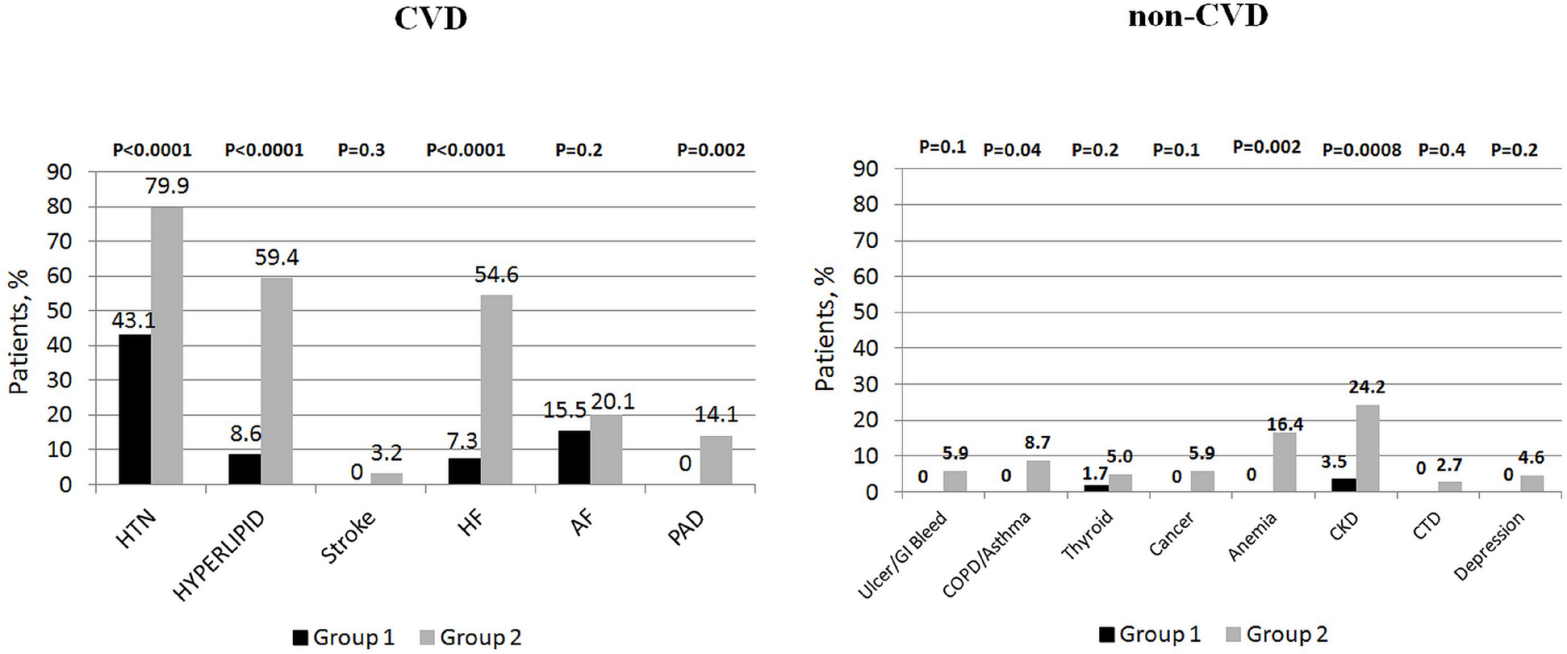
The prevalence of selected cardiovascular (CVD) and non-cardiovascular (non-CVD) comorbidities

**Figure 4:**
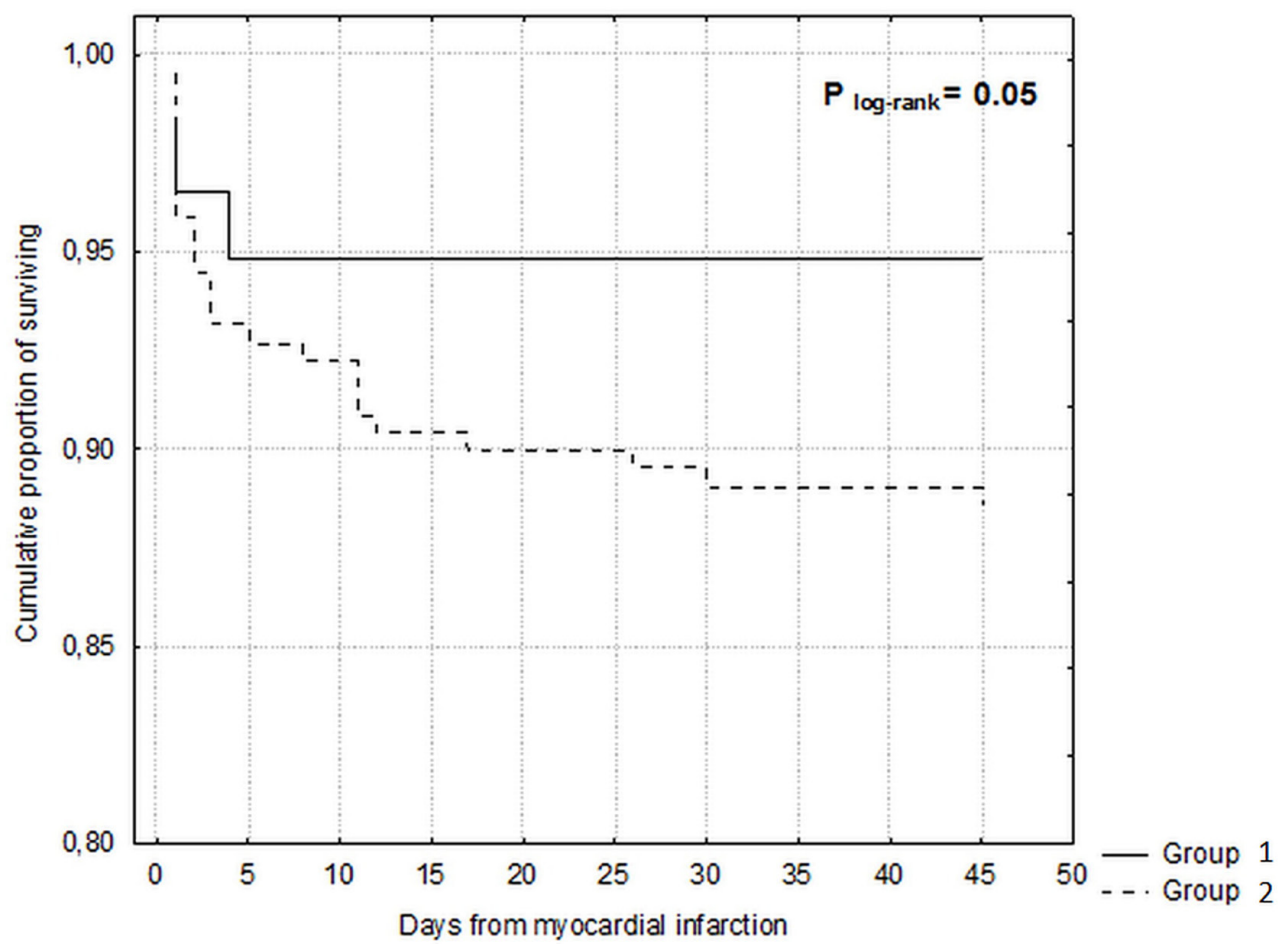
Kaplan-Meier curves for in-hospital survival

**Figure 5:**
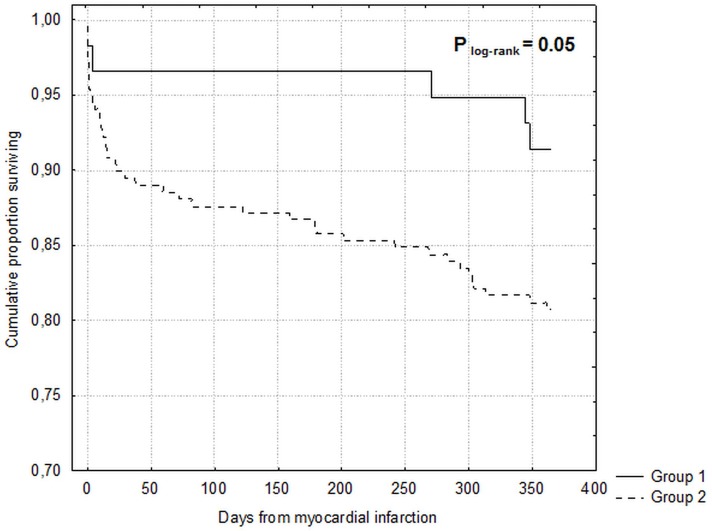
Kaplan-Meier curves for 12-month survival

**Figure 6:**
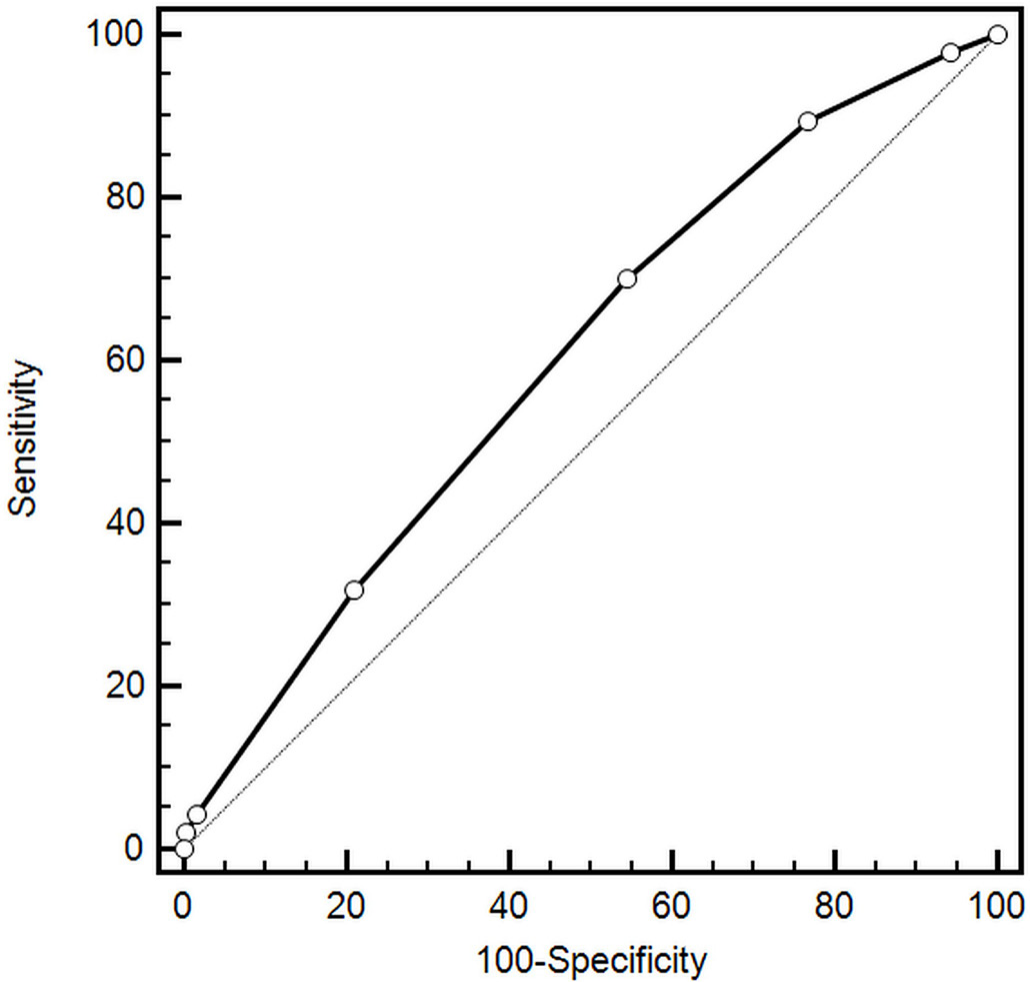
Prognostic value for predicting 12-month mortality

**Figure 7:**
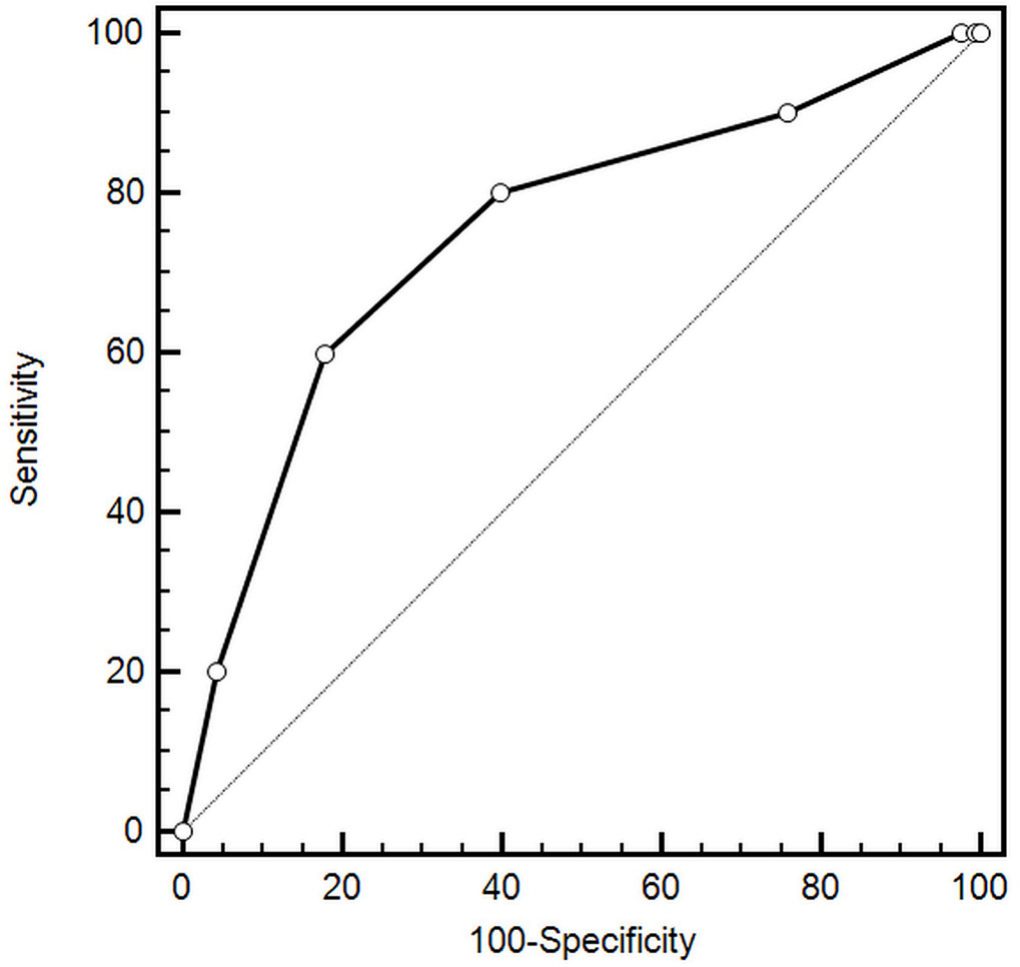
Prognostic value for predicting 12-month acute coronary syndromes

Comorbid conditions were prevalent among study participants (Figure 1). The median number of comorbidities was three. 15.9% of patients had one comorbidity and 22.0%, 34.3%, and 22.7% of patients had two, three or at least four comorbid conditions respectively. The number of comorbid cardiovascular (CVD) and non-cardiovascular (non-CVD) conditions is depicted in Figure 2. A majority of patients had at least one CVD comorbidity (6.1% of patients had none), whereas 53.1% of patients did not have any non-CVD comorbidity. Baseline clinical characteristics are presented in Table 1. The two study groups differed with respect to the use of insulin and metformin. Patients with multimorbidity had more impaired left ventricular systolic function and required longer in-hospital stay (7.5 vs 9 days P=0.04). Figure 3 illustrates the prevalence of selected CVD and non-CVD comorbidities among patients with STEMI and T2DM. Hypertension was the most prevalent CVD comorbidity in group 1, whereas hypertension, hyperlipidemia, and heart failure was the most prevalent CVD comorbidities in group 2. Only small fraction of patients in group 1 had thyroid dysfunction and chronic kidney disease. Meanwhile anemia and chronic kidney disease were the most prevalent comorbidities in the multimorbidity group. Angiographic and laboratory results are summarized in Tables 2 and 3. There were no differences between the two groups in terms of angiographic and laboratory data. During index hospitalization 3 out of 58 patients (5.2%) died in group 1 and 25 of 219 patients (11.4%) died in group 2 (Figure 4). The number of comorbid conditions did not yield a prognostic value for in-hospital mortality in ROC analysis (Table 4). The number of comorbidities was not an independent predictor of in-hospital death (Table 5). During 12-month follow-up, 5 of 58 patients (8.6%) and 42 of 219 patients (19.9%) died, respectively in group 1 and 2 (P=0.05) (Table 6 and Figure 5). Myocardial infarction was more prevalent during follow-up in the multimorbidity group and the rate of stroke was similar (Table 6). The number of comorbid conditions proved modest prognostic value for 12-month mortality and good prognostic value for 12-month acute coronary syndromes in ROC analysis (Table 4 and Figure 6 and 7). Marginal increases in the number of comorbid conditions was associated with a 15% increase in the relative risk of 12-month mortality and a 41% increase in the relative risk of 12-month ACS (Table 5).

**Table 1:** Patients’ baseline and clinical characteristics

	Group 1	Group 2	P
	N =58	N= 219	
Age, years (mean ± SD)	63 ± 8	64 ± 10	0.5
Men, N (%)	37 (63.8%)	126 (57.5%)	0.4
Prior myocardial infarction, N (%)	11 (19.0%)	64 (29.3%)	0.1
Smoking, N (%)	12 (20.7%)	26 (11.9%)	0.04
Time from symptom onset, hours [median (interquartile range)]	5.0 (3.0–6.0)	4.0 (3.0–7.0)	0.8
Cardiogenic shock, N (%)	8 (13.8%)	34 (15.5%)	0.7
Insulin^*^, N (%)	24 (41.4%)	138 (63.8%)	0.002
Metformin^*^, N (%)	34 (58.6%)	88 (40.2%)	0.02
Sulfonylureas^*^, N (%)	19 (32.8%)	70 (32.0%)	0.8
HbA1c, %	7.7 (6.9–8.5)	7.5 (6.9–8.0)	0.3
LVEF, % [median (interquartile range)]	47 (45–51)	40 (35–45)	<0.001
Hospital stay, days [median (interquartile range)]	7.5 (6–10)	9 (6–12)	0.04

SD, standard deviation; LVEF, left ventricular ejection fraction; ^*^ some patients were on more than one hypoglycemic agent.

**Table 2:** Angiographic findings

	Group 1	Group 2	P
	N = 58	N= 219	
Infarct-related artery			
• LAD, N (%)	23 (39.7%)	107 (48.8%)	0.5
• Cx, N (%)	12 (20.7%)	23 (10.5%)	
• RCA, N (%)	21 (36.2%)	75 (34.2%)	
• Other, N (%)	2 (3.4%)	14 (6.4%)	
Multivessel CAD, N (%)	28 (48.3%)	111 (50.7%)	0.8
Initial TIMI flow, N (%)			
• 0	35 (60.3%)	147 (67.5%)	0.6
• 1	12 (20.7%)	36 (16.5%)	
• 2	11 (19.0%)	36 (16.5%)	
• 3	0 (0%)	0 (0%)	
Final TIMI flow, N (%)			
• 0	2 (3.4%)	13 (5.9%)	0.4
• 1	1 (1.7%)	2 (0.9%)	
• 2	3 (5.2%)	20 (9.1%)	
• 3	89.7 (89.9%)	184 (84.0%)	

**Table 3:** Laboratory findings

	Group 1	Group 2	p
	N = 58	N= 219	
Leukocytes, 10^3^/mm^3^	14.5 ± 4.8	13.9 ± 5.6	0.6
Erythrocytes, 10^6^/mm^3^	4.5 ± 0.5	4.5 ± 0.6	0.9
Hemoglobin, g/dL	14.5 ± 1.3	13.9 ± 1.6	0.4
Hematocrit, %	42 ± 5	41 ± 5	0.5
Platelet count, 10^3^/mm^3^	228 ± 66	217 ± 70	0.7
Admission glycemia, mmol/l	9.7 ± 2.7	9.3 ± 3.8	0.7
Total cholesterol, mmol/l	4.7 (4.4–5.8)	5.7 (4.9–7.1)	0.01
HDL cholesterol, mmol/l	1.4 (1.1–1.7)	1.3 (1.1–1.6)	0.8
LDL cholesterol, mmol/l	3.0 (2.5–3.9)	4.2 (3.2–4.6)	0.01
Triglycerides, mmol/l	1.1 (0.8–1.7)	1.2 (0.9–1.8)	0.7
Serum creatinine, μmol/l	83 (76–101)	89 (77–114)	0.5
eGFR, ml/min per 1.73 m^2^	75 (67–87)	70 (60–85)	0.4

**Table 4:** Receiver operating characteristics curves identifying the discrimination thresholds of the number of comorbid conditions for in-hospital mortality, 12-month mortality and 12-month acute coronary syndromes

	Cut off	AUC	95%CI	Sensitivity	Specificity	PPV	NPV	P
**In-hospital mortality**								
Number of comorbidities	> 1	0.52	0.46–0.66					0.7
**12-month mortality**								
Number of comorbidities	>2	0.60	0.55–0.66	70%	45%	21%	88%	0.01
**12-month acute coronary syndromes**								
Number of comorbidities	>1	0.75	0.69–0.80	60%	82%	14%	97%	0.005

AUC – area under the curve, PPV – positive predictive value, NPV – negative predictive value

**Table 5:** Predictors of in-hospital and twelve-month mortality

In-hospital mortality						
	Unadjusted			Adjusted ^*^		
	HR	95%CI	P	HR	95%CI	P
Number of comorbidities (per 1 condition increment)	1.02	0.75–1.40	0.8			
**12-month mortality**						
Number of comorbidities (per 1 condition increment)	1.34	1.05–1.71	0.02	1.15	1.01–1.35	0.05
**12-month acute coronary syndromes**						
Number of comorbidities (per 1 condition increment)	1.46	1.27–1.79	0.005	1.41	1.25–1.68	0.005

^*^ Adjusted for: age (per 1 year increment), history of myocardial infarction, left ventricular ejection fraction, successful percutaneous coronary intervention in the culprit vessel, cardiogenic shock, time form symptom onset (per 1 hour increment), hypertension, hyperlipidemia, heart failure, anemia, chronic obstructive pulmonary disease, chronic kidney disease

HR – hazard ratio; CI – confidence interval.

**Table 6:** Twelve-month follow-up

	Group 1	Group 2	p
	N =58	N= 219	
All-cause mortality, N (%)	5 (8.6%)	42 (19.9%)	0.05
Non-fatal ACS, N (%)	2 (3.4%)	32 (14.6%)	0.03
Stroke, N (%)	0 (0%)	5 (2.9%)	0.2
MACCE, N (%)	7 (12.0%)	70 (31.9%)	<0.01

ACS – acute coronary syndrome, MACCE – major adverse cardiac and cerebrovascular events.

## DISCUSSION

In the current study we have focused on the role of multimorbidity in patients with T2DM and STEMI. There are several key findings of the study. First and foremost, comorbid conditions are highly prevalent among this population of patients. Second, majority of patients have at least 2 other cardiovascular comorbidities and almost half of the patients had one or two non-cardiovascular comorbidities. Nevertheless, despite worse in-hospital outcomes multimorbidity was not an independent risk factor of in-hospital mortality. In terms of long-term follow-up, multimorbidity was associated with worse outcomes. The risk of both long-term mortality and ACS increased with the increasing number of comorbidities.

The presence of multiple comorbid conditions among T2DM patients with STEMI was high. Majority of patients have at least 2 other cardiovascular comorbidities and almost half of the patients had one or two non-cardiovascular comorbidities. Reasons for increased multimorbidity include older age and the presence of T2DM. The largest burden on multimorbidity in T2DM can be attributed to cardiovascular disease, metabolic/endocrine/nutritional conditions and kidney disease [7, 16]. Both the median number of comorbidities as well as the prevalence of multimorbidity was similar to that described in other studies [12, 14, 15, 17]. In our study, hypertension was the most prevalent comorbidity (72.2% in the whole population), followed by hyperlipidemia (48.7%) and heart failure (45.0%). Our results are consistent with data from other studies where the prevalence of hypertension ranged from 50% to 75% [12, 13, 17]. In terms of non-CVD comorbidities, we found that CKD, anemia, and COPD were most prevalent. This, in part, supports earlier findings [14, 18]. However, in contrast to our findings others report depression [18] and GI bleeding/ulcer [19] to be diagnosed in a significant proportion of the studied population. Radovanovic et al studied over 30 000 patients who were enrolled into the AMIS Plus registry and found that 7%, 6%, and 2% of patients were reported to have kidney disease, lung disease, and GI ulcer respectively [15]. Gili et al investigated the effect of comorbidities on in-hospital mortality of more than 5 000 patients with AMI and reported the rates of kidney disease, lung disease, depression, and GI ulcer to be 11%, 16%, 4%, and 0.5% [17].

When considering clinical profile, multimorid patients had more impaired left ventricular systolic function, a longer in-hospital stay, and showed a trend towards a higher prevalence of prior MI (P=0.1). In addition, the use of insulin was higher and metformin was lower in this group of patients. Interestingly, the rate of cardiogenic shock, multivessel coronary artery disease, and successful PCI was similar in both study groups. However, we noticed only a trend towards a higher in-hospital rate in patients with multiple comorbidities (5.2% vs 11.4% P=0.1). Moreover, the number of comorbidities was not an independent risk factor of in-hospital death (adjusted HR 1.02; 95%CI: 0.75–1.40). This finding is contrary to that observed in other studies. Radovanovic et al validated Charlson Comorbidity Index (CCI) in 29 620 patients hospitalized with acute coronary syndromes [15]. CCI was an independent predictor of in-hospital death: CCI1 (OR 1.36; 95%CI: 1.16–1.60), CCS 2 (OR 1.65; 95%CI: 1.38–1.97), and CCS ≥3 (OR 2.20; 95CI: 1.86–2.57). Chen et al 2,972 patients hospitalized with AMI at all eleven greater Worcester medical centers in central Massachusetts and concluded that patients with four or more cardiac comorbidities were more than twice as likely to have died during hospitalization compared to those without any cardiac comorbidities. Moreover, patients with three or more noncardiac comorbidities had markedly increased odds of dying during hospitalization compared to those with no noncardiac comorbidities previously diagnosed [14]. In contrast to the previously mentioned analyses, but similarly to our study, Nguyen et al found similar in-hospital mortality rates irrespective of the number of comorbid conditions 6.8%, 5.5%, and 9.7% for 0, 1, and 2 or more comorbidities respectively P=0.53) [19]. Possible explanations for the lack of impact of multimorbidity on in-hospital mortality in our cohort include: i) the effect of T2DM on in-hospital mortality, (ii) similar age, (iii) similar rate of cardiogenic shock and multivessel coronary artery disease, (iv) similar time from symptom onset, and (v) similar rate of successful PCI among others.

In contrast to in-hospital outcomes, we found a significant impact of multimorbidity on long-term outcomes. Increase in the number of comorbidities by one condition was associated with a 15% increase in the relative risk of 12-month mortality and a 41% increase in the relative risk of 12-month acute coronary syndromes. These results support those observed in previous studies [13, 15]. Available data indicate that co-occurrence of multiple comorbid conditions exert a higher burden on long-term health of MI patients when they do occur separately [13, 20, 21]. The study of Wolff et al further supports the idea of poor outcomes in patients with multimorbidity [5]. Analysis of more than 100 000 Medicare patients indicated that the odds of clinical complications increased exponentially with the increasing number of comorbidities (1 comorbidity was associated with 4 complications per 1 000 beneficiaries; 4 comorbidities was associated with 34 complications per 1 000 beneficiaries) [5].

### Strengths and limitations

Our study should be interpreted in the context of its limitations. The study was carried out among patients with T2DM. As it frequently is a major component of multimorbidity, our results should be interpreted with caution in terms of general population. The small number of patients without multimorbidity could have contributed to the underdetection of meaningful differences in some of the patients’ characteristics, hospital management and short-term outcomes. In addition, we have not registered information on several patient-associated features (e.g., socio-economic status or psychological factors) which may have influenced some of the observed associations. Nevertheless, this is an all-comers study among a high-risk population of patients with T2DM and STEMI.

## MATERIALS AND METHODS

The study conforms to the Declaration of Helsinki. Informed consent for data analysis was obtained from the patients according to the Polish law on patients’ rights regarding data registration. Approval for analyzing recorded data was waived by the local bioethics committee on human research given the retrospective nature of the study. Patients with T2DM admitted with diagnosis of STEMI, within 12 hours from symptom onset were enrolled in the study. This is a single-centre, cross-sectional, retrospective study.

We reviewed the medical records of patients who were admitted with a diagnosis of STEMI and reviewed each of their hospital charts. Comorbidities in the present study were defined as those chronic conditions that were previously diagnosed, and had been documented, in the medical history section of reviewed hospital charts, or that may have been newly diagnosed during the patient’s hospital stay. Comorbidities were grouped into two categories: (a) CVD comorbidities which included hypertension (HTN), atrial fibrillation (AF), heart failure (HF), hyperlipidemia, stroke, and peripheral artery disease (PAD); and (b) non-CVD criteria which included chronic obstructive pulmonary disease (COPD), asthma, cancer, anemia, peptic ulcer/GI bleeding, chronic kidney disease (CKD) stage ≥ 3 (estimated glomerular filtration rate below 60 mL/min/1.73 m^2^), thyroid disorders (hypo-/hyperthyroidism, goiter), depression, and connective tissue disease (CTD). The afore-mentioned CVD and non-CVD were selected based on the findings of previous reports that have pointed to the association of these conditions with outcomes following AMI [12–14, 19].

We adopted the most widely used definition of multimorbidity – that is, the co-existence of multiple chronic diseases and medical conditions in the same individual (defined as two or more conditions) [1, 22, 23]. We used the World Health Organization definition of chronic disease, which is “health problems that require ongoing management over a period of years or decades” [24].

A total of 277 patients with T2DM and STEMI undergoing primary percutaneous coronary intervention (PCI) were enrolled. Based on the number of comorbidities the study population was divided into two groups: group 1 (N=58) with ≤ 1 comorbidity and group 2 (N=219) with ≥ 2 comorbidities.

All patients received loading doses of antiplatelet medications (aspirin, clopidogrel) before admission to our hospital (either in the referring hospital or ambulance) according to the guidelines. Diabetes mellitus was defined as: (a) pre-existing condition diagnosed before STEMI (patients on insulin, oral glucose-lowering drugs or on a diet), (b) newly diagnosed diabetes mellitus based on fasting plasma glucose (FPG) ≥ 7.0 mmol/L or 2-hour plasma glucose ≥ 11.1 mmol/L during an oral glucose tolerance test (OGTT) [25]. To avoid acute hyperglycaemia, FPG was taken into consideration after the third day of hospital stay. For that reason, OGTT was performed on day four of hospital stay or later. STEMI was defined as: 1) ST-segment elevation consistent with MI of at least 2 mm in contiguous precordial leads and/or ST-segment elevation of at least 1 mm in two or more limb leads or new left bundle branch block, and 2) positive cardiac necrosis markers: CK-MB mass (upper limit of normal: 4.9 mg/mL) and/or troponin T (upper limit of normal: 0.014 ng/mL). Patients received 300 mg of acetylsalicylic acid (ASA) loading dose and 600 mg of clopidogrel loading dose, followed by 75 mg of ASA maintenance dose and 75 mg of clopidogrel maintenance dose [26]. Coronary angiography and percutaneous coronary interventions were performed using standard protocols and guidelines. A culprit lesion was described in the presence of an acute occlusion, intraluminal filling defects (or thrombus), ulcerated plaques, dissection, or intraluminal flaps. Successful PCI was defined as a post-procedural residual-diameter stenosis < 30%, with TIMI 3 flow in the infarct-related artery and no procedural complications.

All patients were scheduled for an elective 12-month clinical follow-up. We clinically monitored the patients for cardiovascular events. The major adverse cardiac and cerebrovascular events (MACCEs) included death, rehospitalisation for myocardial infarction, and stroke.

### Statistical analysis

Quantitative data are presented as means ± standard deviations (SD) or medians with interquartile ranges (lower and upper quartiles). Qualitative data are presented as frequencies. The Shapiro-Wilk test was used to determine whether random samples came from a normal distribution. The chi-square test with Yates’ correction was used to compare categorical variables. The unpaired t-test was used to compare normally-distributed continuous variables between groups. The Mann-Whitney U-test was used to compare continuous variables with a distribution other than normal. In-hospital and one-year survival was estimated with the Kaplan-Meier method and compared with the log-rank test. Receiver operating characteristic (ROC) curves were estimated for the number of comorbid conditions. A ROC analysis was planned to identify possible cut-offs to predict and 12-month mortality and 12-month acute coronary syndrome incidence. All clinical variables and laboratory findings with a P value of ≤ 0.05 in the univariate analysis entered into the multivariate Cox proportional hazard survival model using a Wald statistic backward stepwise selection. Multivariate analysis was performed to estimate hazard ratios (HR) and 95% confidence intervals (95% CI) to identify independent predictors of 12-month mortality and acute coronary syndrome incidence while adjusting for potential confounders. A value of two-tailed P < 0.05 was considered significant.

## CONCLUSIONS

Comorbid conditions are highly prevalent among this population of patients. Majority of patients have at least 2 other cardiovascular comorbidities and one or two non-cardiovascular comorbidities. Multimorbidity among T2DM patients with STEMI had no major impact on clinical presentation. The rates of hemodynamic instability, multivessel coronary artery disease and successful PCI were similar irrespective of the presence of multiple comorbidities. In terms of long-term follow-up, multimorbidity was associated with worse outcomes. The risk of both long-term-mortality and ACS increased with the increasing number of comorbidities. In summary, our findings highlight the importance of indentifying patients with multimorbidity. This, in turn, could allow for provision of better care to these high-risk and complex group of patients.

## Abbreviations

ACS –: acute coronary syndrome
AF –: atrial fibrillation
AMI –: acute myocardial infarction
ASA –: acetylsalicylic acid
CCI –: Charlson comorbidity index
CKD –: chronic kidney disease
COPD –: chronic obstructive pulmonary disease
CTD –: connective tissue disease
CVD –: cardiovascular disease
GI –: gastro-intestinal
HF –: heart failure
HTN -: hypertension
MACCE –: major adverse cardiac and cerebrovascular event
OGTT –: oral glucose tolerance test
PAD –: peripheral artery disease
PCI –: percutaneous coronary intervention
STEMI –: ST-elevation myocardial infarction
T2DM –: type 2 diabetes mellitus
